# Effectiveness and safety of ear acupuncture for tension-type headache: A protocol for a systematic review and meta-analysis

**DOI:** 10.1097/MD.0000000000031826

**Published:** 2022-12-02

**Authors:** Fei Lou, Qihui Lou, Jingjing Li, Xiaoli Zhang, Wei Wang

**Affiliations:** a Fuyang District Hospital of Traditional Chinese Medicine, Hangzhou, Zhejiang, China.

**Keywords:** analysis, ear acupuncture, meta, tension, type headache (TTH)

## Abstract

Tension-type headache (TTH) refers to a type of functionalism disease, which is commonly characterized by recurrent headaches of mild to moderate intensity, bilateral location, pressing or tightening quality, and no aggravation by routine physical activity. Has significantly impacts on people’s work and life. Ear acupuncture as a traditional Chinese therapy, showing several advantages (e.g., safety, economy, and less side effects), has been extensively used to treat TTH. However, its curative effect is supported by limited evidence. Accordingly, the present study aims to comprehensively assess the reliability of ear acupuncture in TTH.

**Methods and analysis:**

The study will conduct a systematic review and meta-analysis. Seven databases, including the Embase, Cochrane Library, Pubmed, SinoMed, CNKI, VIP, and Wanfang Data, will be searched using predefined search terms to identify relevant studies. The primary outcomes will be the pain intensity, the pain frequency and the impact of headache. The methodological quality of the included studies will be assessed with a previously established checklist. The Cochrane Collaboration’s bias risk tool will be used for assessing the bias of included RCTs. Stata 17.0 software is used for meta-analysis.

## 1. Introduction

Tension-type headache (TTH) is the most prevalent neurological disorder worldwide and is characterized by recurrent headaches of mild to moderate intensity, bilateral location, pressing or tightening quality, and no aggravation by routine physical activity.^[1,2]^ In the general adult population, 40% to 80% of persons suffer from TTH,^[3–5]^ and also considered common in children and adolescents.^[6]^ Several reports have indicated that 12% of people with TTH lost their jobs because of episodic headaches, and approximately 38% of TTH patients had limitations in regular activities.^[7,8]^ Additionally, TTH both very common and highly burdensome in children and adolescents.^[9]^

The existing treatment of TTH largely complies with drug therapy. Guidelines from the European Federation of Neurologic Societies found that the over-the-counter treatments acetaminophen, aspirin, ibuprofen, and naproxen had level A evidence (effective) for acute treatment of TTH, as did the prescription nonsteroidal antiinflammatory drugs ketoprofen and diclofenac.^[10]^ There are no guidelines to suggest when prevention should be started for TTH. It seems reasonable to consider starting prevention when headache frequency reaches 2 days a week or more, but this largely depends on the level of disability associated with the headaches and patient preference.^[11]^ In addition, adverse drug reactions remain a significant challenge in these drug therapy.^[12,13]^ Thus, a safe and inexpensive treatment with no apparent adverse reactions should be found.

Ear acupuncture is a type of Chinese medicine therapy which is widely used as a complementary and alternative treatment for patients with TTH.^[14–16]^ As a conventional non-drug therapy, it is capable of regulating Yin and Yang, activating blood circulation, and removing blood stasis. Modern studies show that, ear acupuncture has the functions of regulating endocrine, improving immunity and anti-allergy and so on.^[17]^ Previous studies have shown that ear acupuncture is also effective and safe for TTH.^[18]^ But it is still lack of rigorously designed systematic review and meta-analysis on ear acupuncture in the treatment of TTH. This review aims to assess the efficacy and safety of ear acupuncture in TTH treatment at home and abroad to provide evidence-based medicine for clinical practice.

## 2. Methods

### 2.1. Study registration

This protocol was developed according to the guidelines of the Cochrane Handbook for systematic reviews of interventions.^[19]^ It is registered on the International Prospective Register of Systematic Reviews (registration number CRD42022365011).

### 2.2. Patient and public involvement

No patient involved.

### 2.3. Inclusion criteria for collection of studies

#### 2.3..1. Type of study.

It will include all randomized controlled trials (RCTs) of ear acupuncture for TTH, regardless of language or publication status. To be specific, animal trials, case studies, nonRCTs, empirical reports, and reviews will be excluded.

#### 2.3..2. Type of participants.

According to the standardized classification system, the participants were individuals diagnosed with episodic or chronic TTH. No restrictions were imposed on the age or gender of the patients, the course of the disease, or the source of the cases.

#### 2.3..3. Type of interventions.

Intervention measures should be ear acupuncture alone or combined with other methods to treat TTH. If combined with other methods, only the control group with the same intervention measures as the experimental group will be included.

#### 2.3..4. Type of comparator (S)/control.

None of the restrictions on the treatment options for the control group, including no therapy, placebo, or any other control considered for comparison.

#### 2.3..5. Types of outcome measurements.

Studies were considered eligible if they analyzed at least one of the primary outcome measures of interest: pain intensity, pain frequency and impact of headache. These outcomes were selected because they represent the main complaints of subjects with TTH.

### 2.4. Search strategies for recognizing studies

1. The primary source of data.

RCTs of ear acupuncture for TTH will be searched till October 2022 from the Chinese Biomedical Literature Database, Chongqing VIP Database for Chinese Technical Periodicals, China National Knowledge Infrastructure, Wanfang, Web of Science, Cochrane Library, PubMed, and EMBASE. The retrieval strategies adopted by PubMed are elucidated in Table 1.

**Table 1:** Search strategy (PubMed).

Number	Search terms
#1	MeSH: “tension-type headache”
#2	Ti/Ab: “tension-type headache” OR “tension headache” OR “tonic headache ” OR “stress headache” OR “idiopathic headache” OR “psychogenic headache”
#3	#1 OR #2
#4	MeSH: “ear acupuncture”
#5	Ti/Ab: “ear acupuncture” OR “auriculotherapy” OR “auricular therapy” OR “auricular acupuncture” OR “ear acupressure” OR “auricular acupressure” OR “auricular point” OR “ear point”
#6	#4 OR #5
#7	MeSH: “randomized controlled trial” OR “randomized controlled trial as Topic” OR “controlled clinical trial”
#8	Ti/Ab: “randomized controlled trial” OR “random allocation” OR “allocation” OR “haphazard” OR “RCT randomized controlled” OR “randomized” OR “controlled” OR “clinical trial”
#9	#7 OR #8
#10	#3 AND #6 AND #9

Ab = abstract, Ti = title.

1. Search of other resources.

Some unfinished or unpublished experimental data were retrieved from the Chinese Clinical Trial Registry and The Clinicaltrials.gov.

### 2.5. Data acquisition and analysis

**Figure 1.**
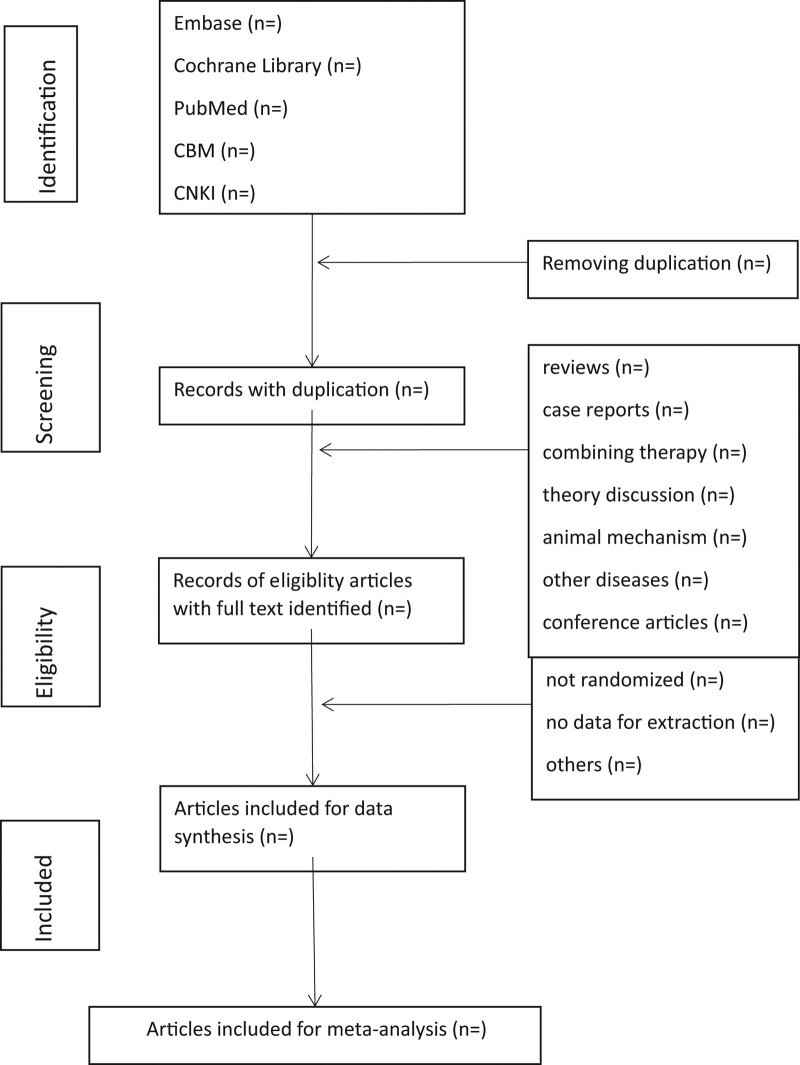
Flowchart of literature selection.

First, all the literature was imported into the EndNote X9 software, and all duplicate literature will be deleted. Second, LF and LQH will be adopted to review the titles and abstracts, and the irrelevant literature will be removed. Third, the full text will be read to determine if the project will be included here. Lastly, 2 researchers (ZXL and LJJ) will conduct the crosscheck. If there were disagreements, the third researcher (WW) would participate in the discussion and solve it. Figure 1 illustrates a flow chart of literature screening.

### 2.6. Data acquisition and management

Two researchers (LF and LQH) will each extract the qualified data into a pre-made table, and a third will step in to resolve any potential differences. The extracted data consisted of journal, author information, title, publication date, participant characteristics, sample size, interventions, study methods, primary and secondary outcome measures, as well as any adverse events.

### 2.7. Assessment of risk of bias in included studies

LF and LQH will employ the Cochrane Bias risk Assessment tool to determine the quality of the trials, respectively.^[20]^ The extracted details were as follows: the random sequence generation, the blindness of result evaluation, the blindness of participants and personnel, the concealment of allocation, the reporting of selective results, the incomplete result data, and so on. These fell to 3 levels, that is, fuzzy, low, and high. In case of ambiguity, the author of the relevant project would be contacted. If there were any disputes, an informed decision would be made with the assistance of the third investigator (ZXL).

### 2.8. Dealing with missing data

In the event of ambiguous data, we will contact the corresponding author by phone or email. We will exclude missing information from the analysis if missing information is unavailable.

### 2.9. Data synthesis and analysis

This study will use Stata 17.0 software for data integration and analysis. The measurement data will use the mean difference (MD) as the effect indicator, and the count data will use the odds ratio (OR) as the effect index. Each effect indicator will be given as a point estimate with 95% confidence interval. The heterogeneity and size of each study result will be judged using statistical methods. For studies with no statistical heterogeneity, the analysis will be performed using a fixed-effect model, whereas a randomized effects model will be applied if for studies with significant statistical heterogeneity.

### 2.10. Subgroup analysis

If there were significant heterogeneity between the trials involved, the course and sample size, type, time, and frequency of ear acupuncture would be considered for subgroup analysis.

### 2.11. Sensitivity analysis

Sensitivity analysis was conducted to exclude low-quality literature to ensure the stability and accuracy of the conclusions drawn from this meta-analysis.

### 2.12. Assessment of reporting biases

If the number of RCTs exceeded 10, funnel plot analysis would be required to test for publication bias. In addition, if there was an asymmetric funnel graph, the Egger check would be conducted to study the causes of publication bias.

### 2.13. Ethics and dissemination

This study will not involve primary data collection, and formal ethics approval will, therefore, not be required. The results from this study will be disseminated through conferences and in peer-reviewed journals.

## 3. Discussion

TTH is the most common primary headache, with a high prevalence and a considerable socioeconomic impact.^[21]^ Drug therapies are effective in treating TTH, whereas there are concerns about the side effects of drug therapies. In contrast, ear acupuncture, as an effective technique of TCM, has been accepted for TTH treatment in China. However, as far as the current study is concerned, the efficacy and safety of ear acupuncture in TTH treatment are not supported by data. Thus, this study is expected to evidence the clinical use of ear acupuncture in TTH treatment.

Some possible limitations of this study should be claimed here, including poor quality of the original study, false positive or negative results, different disease duration, different intervention doses, different intervention frequencies, language limitations, etc. These limitations may lead to certain biases and affect the evaluation results.

## Author contributions

All authors have read and approved the publication of the protocol.

**Conceptualization:** Fei Lou.

**Data curation:** Fei Lou, Qihui Lou, Xiaoli Zhang, Jingjing Li, Wei Wang.

**Formal analysis:** Fei Lou, Qihui Lou.

**Investigation:** Fei Lou, Xiaoli Zhang, Jingjing Li.

**Methodology:** Fei Lou, Xiaoli Zhang, Wei Wang.

**Software:** Jingjing Li, Wei Wang.

**Supervision:** Fei Lou.

**Writing – original draft:** Fei Lou, Qihui Lou, Xiaoli Zhang, Jingjing Li.

**Writing – review and editing:** Fei Lou, Wei Wang, Qihui Lou, Xiaoli Zhang.
